# Religious Change over the Generations in an Extremely Secular Society: The Case of Sweden

**DOI:** 10.1007/s13644-017-0294-5

**Published:** 2017-05-11

**Authors:** Magnus Hagevi

**Affiliations:** Department of Political Science, Linnaeus University, 351 95 Växjö, Sweden

**Keywords:** Generation, Religious change, Secularization, Religious market, Salvation, Existential security

## Abstract

The main argument of this paper is that religious change caused by modifying supply in the religious market takes time due to intergenerational value change. Unlike previous research, this study suggests that not only do religious agents on the supply side need time to adjust, but that the time lag is probably even greater among individuals on the demand side of the religious market. Using time series data, the study demonstrates that, despite shrinking church attendance, interest in religious concepts such as “salvation” has increased in the generations born after 1970 in Sweden. Describing the transformation of the Swedish religious market from a regulated religious monopoly before 1970 to an increasingly deregulated and competitive religious supply since 1970, the study explains this revival in religious interest on the part of generations whose formative years were after 1970. As these generations replace previous generation with less religious interest, religious interest is rising in the Swedish population. The conclusion holds even when controlling for period and lifecycle effects, as well as alternative explanations of religious change such as increased migration and the existential security thesis.

The strong and ongoing secularization of religiously deregulated but extremely secular societies (Bruce 2000) is a major problem for the controversial religious market theory (Iannaccone 1997; Stark and Finke 2000). In highly secular Northern European societies such as that of Sweden (Inglehart and Welzel 2005), despite a deregulated religious market like that created when the Swedish government profoundly altered its relationship with the established Church of Sweden (Pettersson 2011), secularization seems to be ongoing or faces ceiling effects (Burkimsher 2014). However, I argue that religious changes caused by modifying supply in the religious market may need some time to occur (Aarts et al. 2010). In contrast to previous findings, I suggest that it is not only the religious agents on the supply side who need time to adjust, but that the time lag is probably even greater for religious demand among individuals on the demand side of the religious market. In this paper, religious change on the demand side is strongly linked to intergenerational value change (Inglehart and Welzel 2005). I also argue that changes in religious supply should not only, and not even mainly, be sought in the dominant denomination’s statutory relationship with the state, but also in this denomination’s shrinking dominance in the everyday lives of people.

It is unsatisfactory that previous research into extremely secular societies (Burkimsher 2014; Hagevi 2005; Skårhøj and Østergaard 2005; Voas and David 2010) has noted increased interest in religion, but failed to theoretically explain this shift. Referring to religious market theory (Iannaccone 1997; Stark and Finke 2000) and emphasizing different socialization during the formative years of distinctive generations (Inglehart and Welzel 2005; Zukin et al. 2006), I argue, partly contrary to previous research (Ruiter and van Tubergen 2009), that interest in religion will increase in an extremely secular society.

My main argument is that changes in religious supply affect how different generations express their religious demand. Describing the transformation of the Swedish religious market from a regulated religious monopoly (Stark and Finke 2000) to an increasingly deregulated religious supply in a more competitive religious market, this study explains this revival in religious interest. The concept of religious interest refers to increased cognitive (psychological) attention without being bound to changed behavior. To put my proposed explanation to a critical test, the study will control for period and lifecycle effects, as well as for the market theory alternative explanations of religious change such as increased migration from less secular countries than Sweden (Ruiter and van Tubergen 2009; Voas and David 2010) and Pippa Norris and Ronald Inglehart’s (2004) revised modernization theory, here called the existential security thesis.

To explore a society with an extremely high level of secularization, the study focuses on Sweden, a Northern European country with an extremely secular and individualized population (Inglehart and Welzel 2005) in a traditional protestant (Evangelical-Lutheran) state church tradition. Since 2000, the Church of Sweden claims to be independent of the government, but still the government collects the member fees of the Church of Sweden (Pettersson 2011). As for the theory of religious change in extremely secular societies, Sweden is a critical case. If the theory is not supported in a Swedish context, it would probably not receive confirmation if it were to be tested on other cases. Scholars of religious market theory, as well as those of modernization theory, portray Sweden as an archetype for a highly secular society (Inglehart and Welzel 2005; Stark and Finke 2000). However, Ulrich Beck (2010) noted what he calls *the paradox of secularization*, which results in increased religious interest. He outlines individualized and secular societies, like Sweden, as a setting for the disempowerment of religious organizations—such as the Church of Sweden—as well as for the possibility of individual religiosity.^1^ Beck assumes that with individualization and secularization, religion has been freed from legitimizing the government’s political power and may now devote itself to spirituality.^2^ This process is infused by the erosion of clear boundaries separating societies, cultures and religions. I argue that this development has deregulated the supply and stimulated the demand in the Swedish religious market.

This paper is organized as follows. The next section presents hypotheses concerning religious change in an extremely secular society, which is followed by a presentation of why lifecycle, time period, immigration, and existential security may affect religious change across generations. The following section briefly surveys previous research. The paper then describes the data and the research design. In the empirical sections, the paper first presents the modified religious supply and then the observed intergenerational changes in religious interest. At the end, the study presents its conclusions.

## Theory of Religious Change in Extremely Secular Societies

### Religious Market

Religious market theory (Stark and Finke 2000) distinguishes between religious supply and demand. Religious *supply* concerns religious beliefs, belonging and conduct offered by religious institutions, whereas religious *demand* refers to religiosity among individuals. A basic assumption is that deregulated religious markets (i.e., religious freedom) with a competitive and pluralistic supply (i.e., many competing religious organizations) tend to satisfy the religious demand better compare to religious monopolies (i.e., a dominant church supported by government regulations). While an open and competitive religious market stimulates religious interest, a religious monopoly increases secularization. In extremely secular societies—as in Sweden—even fairly small changes in religious pluralism can increase religious competition (Hamberg and Pettersson 1994), possibly because weak religious ties tend to reduce the cost to individuals of religious change (cf. Iannaccone 1997).

### Changes in Religious Supply

If religious regulations force people to conform to religious norms and conduct, compulsory religion may socialize people to avoid religion when possible. If such a religious market is deregulated and religious conducts and norms become optional, those who want to avoid religion will probably take the opportunity to be non-religious. However, when religion is deregulated “religion is forced to be religion and nothing else” (Beck 2010: 25). This is a new religious supply. Individuals with no experience of the previous regulated religious market may use this option without the experience of compulsory religious conduct that resulted in a negative opinion of religion. These individuals, at least compared to individuals who experienced compulsory religion, may show greater religious interest. When a religious market changes from a monopoly to a free and competitive market, not only might religious behavior change; the general interest in religion may also increase.

Regulation concerns *formal* and *informal* religious norms in society. *Formally*, religion may be integrated with the state: deregulation would then concern separation between church and state (Berger 1967). The withdrawal of the government from fostering citizens in religion via the curriculum of grade schools differentiates church and state, and is used as an indication of religious market deregulation. As childhood socialization during the formative years of the individual has proved important (Ruiter and van Tubergen 2009), the secularization of religious studies in grade school directly affects the individual.

*Informal* religious norms affecting individuals can be evoked by churches. Such informal norms are an important part of a religiously regulated society that directly affects people in their daily lives. If religious norms are also part of the general norms of society, apart from their personal beliefs, individuals must adhere to informal religious norms. Example of religious norms could be that couples must marry before starting a family. In the public eye, anything else would be “bad behavior”. Social pressure to adhere to religious norms is much lower if they are not part of the general social norms. If the supply of religious institutions fails to influence the surrounding society, traditional informal religious norms will come to mean less to individuals (Berger 1967; Luckmann 1967) and their acceptance may depend on personal interest (Beck 2010). Then, the religious norms are no longer general social norms and, for instance, the level of performed religious rites may decrease. In this study, the percentage of people who perform traditional religious rites implies success in transferring informal religious norms to people (Hervieu-Léger 2006); a high degree of *unperformed* religious rites signals weaker religious norms in society.

Generally, scholars acknowledge that *competition* in the religious market increases with the increase in religious *pluralism* (Chaves and Gorski 2001; Norris and Inglehart 2004; Ruiter and van Tubergen 2009), since religious competition is absent in religious monopolies. Religious pluralism tends to produce “the coexistence of different frequently contradictory world perspectives and value systems in a space where they directly interact” (Beck 2010: 152). New religious products in a market (e.g., Islam in Europe) may stimulate religious interest, not only by creating more available options to satisfy the demand, but also by making otherwise secular people more conscious about religion. When the interactions between Christian, Muslim and secular individuals increase, this will lead to “comparisons between world-pictures of the various religions and discussions of their relevance in everyday life” which “act as a form of existential stimuli” (Beck 2010: 41). Individuals in otherwise religious passive segments of society get increasingly interested in religion as they reflect on their own and others’ religious identity.

As is claimed here (see below), the Swedish religious market became less formally and informally regulated and more competitive around 1970, which changed how younger generations encountered religion during their formative years compared with the experience of older generations. To the degree that these younger generations display different religious interests from those of the older generations, this will be interpreted as an effect of changes in supply in the religious market.

#### **Hypothesis 1**

The religious market was more deregulated during the formative years of the younger generations than during those of the older generations.

#### **Hypothesis 2**

Religious pluralism was greater during the formative years of the younger generations than during those of the older generations.

### Generations and the Formative Years of the Individual

As well as religious organizations requiring time to adjust to a new situation (Aarts et al. 2010; Stark and Finke 2000), one is likely to find a time lag in individual responses to supply change on the demand side of the religious market. However, this response concerns religious values deeply rooted in the individual. Several researchers (Inglehart and Welzel 2005; Zukin et al. 2006) propose that intergenerational differences and population replacement constitute the basis for such value changes in society. The core assumption for generational effects on religious changes is that each new generation has the potential to develop its own special traits. Among scholars there is a widespread agreement of increased individualization of each new generation in advanced industrial societies. This process means, among other things, that individuals are less loyal to a common identity (Putnam 2000), rebel against authority figures (Bjereld and Demker 2005), have different lifestyles, and value self-expression (Inglehart and Welzel 2005). Such generational changes may give rise to new demands in the religious market, as protests to compulsory religious conduct grow.

Childhood, adolescence and young adulthood are crucial periods for socialization (Iannaccone 1997: 33; Inglehart and Welzel 2005), when the basic identity of the individual and the generation are being formed. The special traits shared by a generation—such as attitudes toward religion—are likely shaped during the constituent individual’s formative years. I suggest that the structure of the religious market during an individual’s formative years is central to the development of lifelong religious interest. If the formative years of a generation coincide with a religious monopoly, this generation will have religious interests compatible with a religious monopoly even if the religious market is later deregulated and becomes more pluralistic. If these individuals belong to a generation that tends to be more individualized than previous generations, they will tend to be less interested in religion than previous generations. Likewise, a relatively individualized generation whose formative years are spent in a freer and more competitive religious market tends to display religious interest affected by this context throughout the life course.

Zukin et al. (2006) identify four recent generations and the central factors creating the experiences of each of these four generations correspond to similar conditions all over the Western world. However, this study uses different labels for the oldest and youngest generations from the one applied by Zukin et al.

The *Pre*-*War Generation* comprises those born before 1946, whose formative years were marked by depression in the Western world, two world wars, and their aftermath. Some describe the members of this generation as relatively materialistic (Inglehart and Welzel 2005), while others emphasize their values of duty, self-sacrifice, and loyalty to collective identities (Zukin et al. 2006). The shared experiences of this generation meant that the religious supply of their time suited this generation fairly well, explaining why their religious interest is relatively high.

The *Baby Boomers* (a sizeable generation in Sweden as in the U.S.) were born in 1946–1964. They often expressed individualism, rebelled against authority, started the sexual revolution, and conducted political protests such as Swedish demonstrations against the U.S. war in Vietnam (Bjereld and Demker 2005). This generation experienced increasing prosperity, in Sweden linked to the expansion of the welfare state (Lindvall 2004). Compared with the previous generation, a relatively large part of Baby Boomers saw religious authorities as representatives of a religious monopoly unwilling to change and as unnecessarily constraining their individual freedom. Religious authorities become figures to rebel against or, as quickly as possible, stop caring about. The Baby Boomers become relatively secular (Bjereld and Demker 2005).

Members of *Generation X* were born between 1965 and 1976, and scholars relate them to a greater individualism than the Baby Boomers (Zukin et al. 2006). This generation’s formative experiences were framed by familial and financial insecurity in the Western world: rising rates of family separation (Surkyn and Lesthaeghe 2004), threat of AIDS (Herlitz and Brorsson 1990), recession, and governments’ economic failures (Lindvall 2004). A large part of Generation X grew up with no religion at all, mainly because their parents, often Baby Boomers, lived truly secular lives, breaking the generational chain for transferring religious norms (Hervieu-Léger 2006). Moreover, the Swedish government had withdrawn from regulating religious supply. Such a change in religious supply was accompanied by additional deregulation of the religious market: religious norms ceased to exist as general and informal social norms; instead, religion became a free choice not only legally but also socially.

Members of *Generation Y* were born after 1976 (Zukin et al. 2006). Scholars have called the context of its formative period hyper-individualized (Bjereld et al. 2005). The communicational revolution is a global mark for this generation, enabling them to find digital communities of interest, not bounded by territory. On top of the deregulating religious market that faced Generation X, Generation Y was part of a society of greater pluralism and competition in the religious market. Immigrants, increasingly originating from regions outside Europe with less secular non-Lutheran cultures, were coming to Sweden (Gustafsson 2000). Subject to these stimuli in their formative years, members of Generation Y ought to display greater interest in religion than do Generation X or the Baby Boomers.

#### **Hypothesis 3**

Individuals in the Pre-War Generation and Generation Y express greater religious interest than does Generation X, which in turn shows more religious interest than Baby Boomers.

### Lifecycle and Period Effects

To validate the generational effect on religious interest, this study also controls for lifecycle and period effects.

#### **Hypothesis 4**

The religious interest of individuals may temporarily change due to period effects, which, in turn, moderate generational effects on religious interest.

#### **Hypothesis 5**

With increasing age, individuals express a greater interest in religion, which, in turn, may modify generational effects on religious interest.

### Foreign Background

During the period covered by the study, there has been considerable growth in immigration to Sweden. Because the Swedish population is extremely secular, immigrants to Sweden tend to display a comparatively greater interest in religion (Ruiter and van Tubergen 2009; Voas and David 2010). Thus, immigration adds individuals to the religious population in Sweden. To control for this, the study also investigates the impact of individuals of a *foreign background*.

#### **Hypothesis 6**

The increasing share of people of a foreign background explains the generational changes of religious interest.

### Existential Security

Norris and Inglehart (2004) are critical toward the supply-side oriented religious market theory. As an alternative they amended the modernization theory with the existential security thesis in order to also allow for other changes than secularization. Norris and Inglehart emphasize changes in the public demand for religion. According to them, as society modernizes, the feeling that existence is secure enough that it can be taken for granted is a force of secularization, while “feelings of vulnerability to physical, societal and personal risks are key factors directly driving religiosity” (Norris and Inglehart 2014: 3391). The thesis states that religious reassurance is less important for individuals living in existentially secure societies than for individuals living in less existentially secure societies.

This study analyzes the main assumption made by the existential security thesis to critically test the explanations of the market theory. As Generations X and Y are described as more interested in religion than the Baby Boomers, some researchers claim that their formative years were less prosperous than those of the Baby Boomers (Zukin et al. 2006). Therefore, different degrees of existential security may account for some of the religious variance among generations.

#### **Hypothesis 7**

Variations in existential security explain generational changes of interest in religion.

## Previous Studies

Some previous studies conducted complete tests of religious market theory and examined how religious behavior is affected by both religious deregulation and pluralism. These studies reported both positive (Chaves and Cann 1992) and negative results (Verweij et al. 1997) with regard to the impact of both deregulation and competition. One of these studies obtained negative results for religious pluralism but positive results for religious deregulation (Barro and McCleary 2003). Some studies treated religious deregulation and pluralism in separate analyses. For religious deregulation, some report positive results (e.g., Aarts et al. 2010) and others negative results (e.g., Bruce 2000; Norris and Inglehart 2004). Concerning religious pluralism, some report positive results (e.g., Hamberg and Pettersson 1994; Ruiter and van Tubergen 2009) and others negative results (e.g., Chaves and Gorski 2001; Norris and Inglehart 2004).

A single study reflecting in a possible time lag between deregulation and changes in religious behavior (Aarts et al. 2010) found no effects attributable to the duration of religious deregulation. To my knowledge, no study of market theory relates to interest in religion. Some studies noted an increase in religiosity among younger generations in extremely secular societies, among them Sweden (e.g., Burkimsher 2014; Voas and David 2010). Scholars also report positive results from testing the existential security thesis using cross-sectional data (Norris and Inglehart 2004, 2014; Ruiter and van Tubergen 2009).

## Data and Measurement

Since this paper concerns religious change over time, the data must be longitudinal. The study uses several data sources to investigate religious supply, both formal and informal. Firstly, to determine the degree of formal integration between church and state, the study presents a secondary analysis of the status of religious education in *grundskola*, or Swedish grade school (Dahlgren 1985; Gustafsson 2000). This religious education constitutes a form of religious market regulation. Secondly, to gauge the informal regulation of religious supply, the paper examines the importance of informal religious norms in society using data on the number of performed religious rites from various sources, mainly the Church of Sweden and Statistics Sweden, the Swedish government bureau of statistics (Statistiska centralbyrån 1969, 1992, 2013; Stoltz 1970; Svenska kyrkan 2009, 2015). Thirdly, to determine the change in religious pluralism and competition, the study compares the denominational affiliations of the Swedish population in 1930 and 1999 using the national census of 1930 (Statistiska centralbyrån 1937), and a tally of all religious attendance in all religious denominations during a Friday-to-Sunday period in November 1999 supervised by the Church of Sweden’s research unit (Skog 2001). Although these two sources of statistics are not completely compatible, they provide an indication of religious pluralism in Sweden 1930 and 1999.

To investigate individual interest in religion, it would be best to use surveys directly measuring religious interest over a long period. Regrettably, such data are not available, since most longitudinal data cover only religious behavior, do not include important control variables (as foreign background), and have only few points of measure (cf. European Values Study 2015). Instead, the study uses surveys that include a proxy for religious interest: the *importance of salvation*. In the surveys, “Salvation” is one item in a battery of 25 items, with the question “How important do you think the following items are to you?”, originally measuring terminal and instrumental values presented by Milton Rokeach (1973). The response options of the survey uses the Likert-type scale: “Very important” (code = 5), “Somewhat important” (code = 4), “Neither important nor unimportant” (code = 3), “Not very important” (code = 2), and “Not important at all” (code = 1). Depending on the year of the survey, the number of respondents is between n = 1506 and n = 1835. If people regard religious concepts such as salvation as important, this also indicates interest in religion. The more important respondents think salvation is, the greater their interest in religion (and vice versa). Indeed, people can attach quite different religious connotations to the word “salvation”. In this particular context—a survey of a sample of an extremely secular population—a narrow interpretation of the responses is incorrect. Instead, the rated importance of salvation is treated as an indication of interest in one of many possible religious values and concepts. If the rated importance of salvation increases over time, this probably indicates greater general psychological interest in religious phenomena. The study uses data on the importance of salvation from 16 surveys conducted by the SOM Institute, University of Gothenburg, between 1986 and 2011, referring to samples representing the Swedish population 16–85 years of age in 2011, 15–85 years of age in 2000–2008, 15–80 years of age in 1992–1998, and 15–75 years of age in 1986–1991 (Vernersdotter 2012). The changing age range of the sample does not affect the conclusions of the study.

I measure the share of individuals of a foreign background by categorizing people immigrating to Sweden and people with at least one parent who immigrated to Sweden as “foreign background” (compared with “others”) in the surveys conducted by the SOM Institute.

The effect of existential security is measured with an index comprising four items (i.e., terrorism, environmental destruction, economic crisis, and high unemployment) using the following question: “If you consider the current situation, how unsettling would you then perceive the future for each item, respectively?”, with the response options being “very unsettling”, “rather unsettling”, “not so unsettling”, and “not unsettling at all”. The items form a summed index ranging from 0 (insecure) to 1 (secure).^3^

Foreign background and the existential security index were controlled for on a smaller sample because they were not part of all the surveys measuring the importance of salvation.

## Religious Supply

This section illustrates how the change in religious supply altered the Swedish religious market during the formative years of Generations X and Y.

### Religion in Grade Schools

The withdrawal of the government from fostering citizens in religion is a sign of formal religious market deregulation and exemplifies the differentiation of church and state. This section is based on previous research (Dahlgren 1985; Gustafsson 2000) and describes the secularization of religious education in Swedish grade school, from confessional education to the establishment of a non-confessional and mainly social-scientific religion curriculum. The secularization of education displays a change from regulated religious conduct to religion presented as an option according to the will of the individual. The religious education conducted in grade schools of the Pre-War Generation and Baby Boomers was an attempt to socialize them into religious conduct. Since this conduct was controlled, tested and graded by teachers it was compulsory.

Until 1919, the 6-year grade school provided a purely confessional religious education in the teachings of the Church of Sweden, called “Christianity” (about 10 h of instruction a week), with the catechism of Martin Luther serving as the literature. In connection with the democratic breakthrough in Sweden in the early 1920s, thorough reform transformed this religious education into 2 h a week of ethical–evangelical education. The main literature was the Bible, particularly Jesus’ Sermon on the Mount. Into the 1950s, the Church of Sweden also had special rights to scrutinize the “Christianity” instruction in grade schools. In 1962, when grade school was extended to become 9-year compulsory school, “Knowledge of Christianity” (*kristendomskunskap*) replaced the former subject labelled “Christianity”. The purpose was no longer to lay the foundation for a Christian philosophy of life but to orient students toward the Christian faith. In the early 1970s, when the first cohort of Generation X began school, grade school broke with the tradition of Christian religious education. With the major curriculum reform of 1969, the name of the religious field of study changed to “Knowledge of religion” (*religionskunskap*). Under this curriculum, the subject no longer constituted teaching in the Christian faith but became a social science subject, as the teachers taught about all major world religions, although they had to stress Christianity.

Until the late 1950s, schools in general called pupils to compulsory *morning prayers*, with Christian reflection, prayer and hymns. In 1958, the name was changed to *morning assembly* to also incorporate elements of social life and culture, but even at this point they tended to have a Christian character. The major curriculum reforms in the 1960s ended the religious morning assemblies.

The Baby Boomers and the Pre-War Generation received state-controlled teaching in the Christian faith, although to different degrees, which was generally the same as the teaching of the Evangelical Lutheran state church. For an increasing number of pupils, especially among the individualized Baby Boomers, this education was compulsory religious conduct without any relation to personal interest. For these individuals, contrary to the educational purpose, religion probably became something to avoid. The focus on Christianity stopped around 1970, also ending the potentially negative perceptions of religion as a result of compulsory religious conduct in school. The reduced government shaping of religion via school also constituted an important deregulation of the religious market, right at the time Generation X entered grade school. This result supports Hypothesis 1, indicating that, compared with the Baby Boomers and the Pre-War Generation, Generations X and Y were educated in secularized grade school and faced a deregulated religious market during their formative years.

### Importance of Religious Norms in Society

**Fig. 1:**
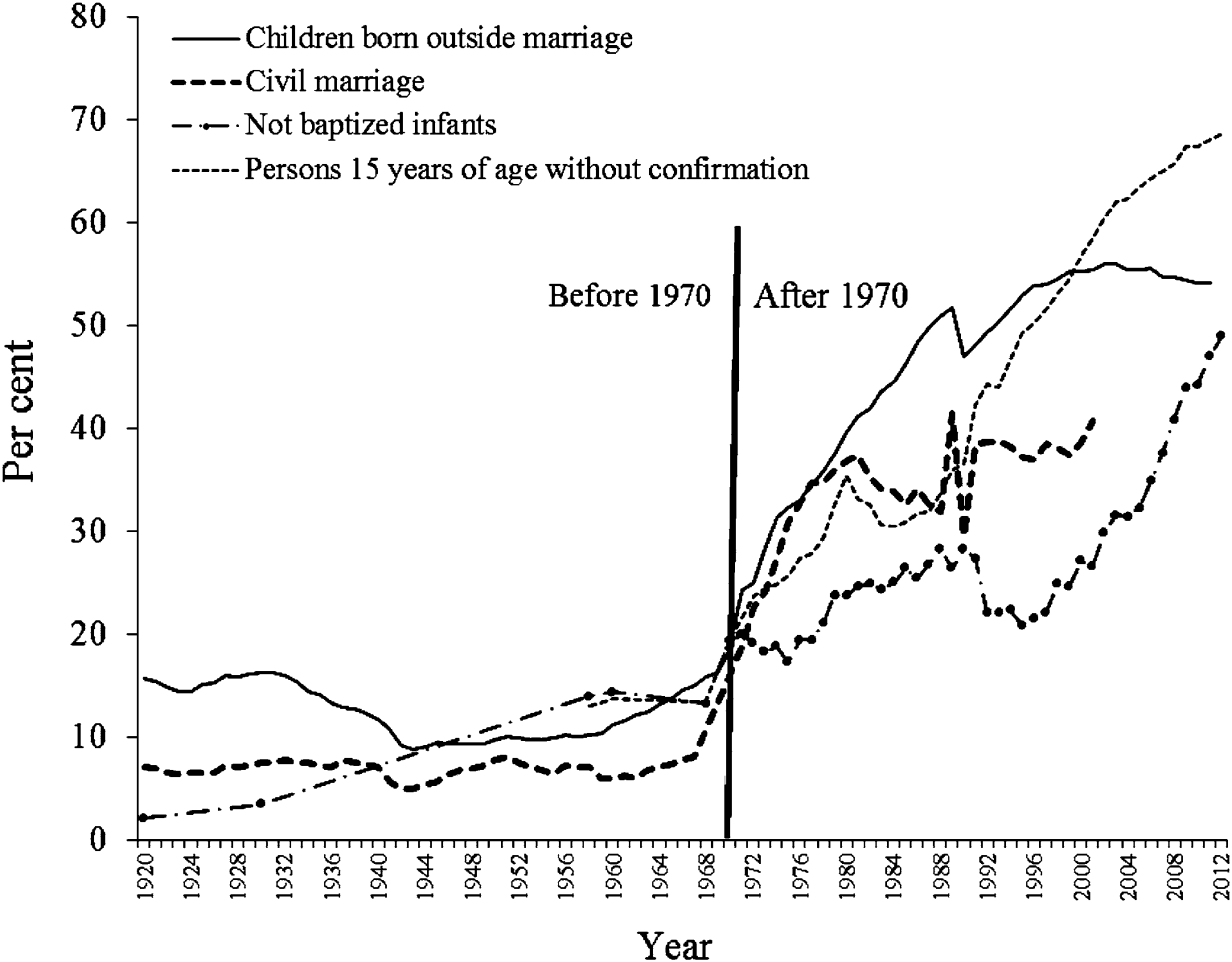
Indications of organizational secularization: unperformed religious rites, 1920–2012, percent. Children born outside marriage are presented as the percent of all live births. Civil marriage is presented as the percent of all marriages. Unconfirmed people 15 years of age are presented as the percent of all people 15 years of age. Unbaptized infants are infants not baptized in the Church of Sweden as a percent of all newborn infants in a given year. *Sources* civil marriages, 1970–2001 (no official records available after 2001), Svenska kyrkan (2009); baptized infants and confirmation, 1970–2014, Svenska kyrkan (2015); baptized infants and confirmation in 1960 and 1968, Stoltz (1970: 22); children born outside marriage, 1920–1967, Statistiska Centralbyrån (1969: 96–99), 1968–1990, Statistiska Centralbyrån (1992: 47), 1991–2007, Statistiska Centralbyrån (2013: 90)

Figure 1 presents indications of the status of informal religious norms in society in the form of four unperformed religious rites. Civil marriages as a proportion of all marriages as well as the proportion of children born outside marriage jointly imply that the religious rite of marriage is not being performed before family formation. The baptism of newborn children and confirmation of 15 year olds are traditional religious rites, since most people in Sweden are members of the Church of Sweden. If such rites are not performed, this indicates a break from important religious norms in society.

Around 1970, there was a dramatic increase in the nonperformance of religious rites. Before 1970, most rites were conducted according to the traditions of religious institutions. Afterward, organized religion lost ground to an increasingly secular society in a transition that in some respects is still ongoing. This religious decline implies looser social norms and the lessening of social pressure for individuals to perform religious rites, meaning that religious norms are no longer the general norms of Swedish society. These data do not mean that individuals in Sweden were dramatically more religious in the 1960s than in the 1970s (Hagevi 2005). Instead, most data suggest that the magnitude of individual secularization may have reached a point at which citizens simply disregarded religious norms and the religious superstructure collapsed by around 1970, as it seemed meaningless to many individuals.

The loss of importance of informal religious norms is another form of religious market deregulation in Sweden that occurred after 1970 during the formative years of Generation X and was taken for granted by most individuals in Generation Y. The Pre-War Generation and the Baby Boomers were both raised in a religious market shaped by socially active religious norms. Among those who were forced to obey these religious norms disregarding their own religiosity, especially among Baby Boomers, negative attitudes toward religion may have grown stronger. However, in accordance with Hypothesis 1, as the formative years of Generations X and Y were in a more deregulated religious market than those of previous generations, they may not developed such negative attitudes toward religion.

### Religious Pluralism

Church of Sweden membership is falling, almost everyone was a member of the state church during the formative years of the Baby Boomers and the Pre-War Generation, but non-church members became increasingly common during the formative years of Generations X and Y. Besides being a sign of secularization, this decrease in religious dominance marks a loss of religious monopoly, necessary to create an opening of the religious market for other religious organizations to increase religious pluralism.

Starting around 1970, more people began immigrating to Sweden, making Sweden more multi-religious during the formative years of Generation X, and even more so of Generation Y, than during the formative years of the Baby Boomers and especially the Pre-War Generation. Furthermore, Protestant free churches have long been active in Sweden (Gustafsson 2000). Indeed, Swedish society is more multi-religious in the twenty-first century than it was in 1930 (Skog 2001; Statistiska Centralbyrån 1937). The Herfindahl index (H) of religious creeds was 0.87 in 1930, indicating high religious concentration; by the turn of the millennium, the concentration had decreased (and religious pluralism increased) as indicated by an H of 0.66.

This growing religious pluralism indicates that the increasing deregulation of the religious market was accompanied by growing religious competition. This stronger competition in the religious market occurred during the formative years of Generation X and even more so for Generation Y, as suggested in Hypothesis 2. Again, an important change took place, mainly between the Baby Boomers and Generation X.

## Religious Demand

**Fig. 2:**
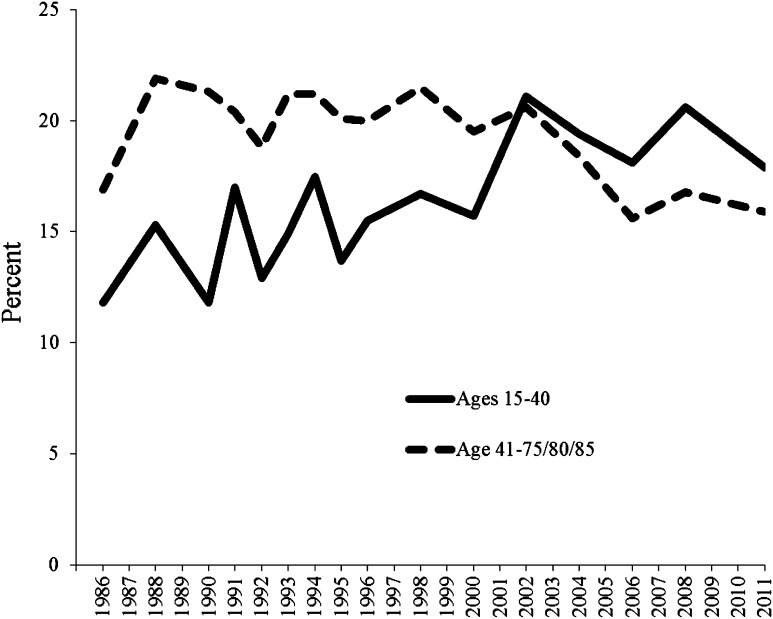
Importance of salvation by age, 1986–2011 (percent very or somewhat important). *Source* SOM Institute, University of Gothenburg

The stated importance of salvation is used as an indicator of religious interest. Figure 2 shows data on the percentage of stated salvation as very or somewhat important, 1986–2011, in two age cohorts: 15–40 years of age and 41–75/80/85 years of age. For both age cohorts, the reported percent is low (between 11 and 22%), which is in accordance with the conception of Sweden as an extremely secular society. According to the assumptions of ongoing secularization (Bruce 2000), salvation should be rated as less important among younger generations than older generations. If so, we would expect salvation to be rated more important among people above 40 years of age and less important among people 40 years old and younger. Indeed, we would expect that both age cohorts would rate salvation less important as time goes by.

The importance of salvation is changing over time in both age cohorts, but not according to the traditional expectations of secularization. In 1986, as suspected, the older age cohort rated salvation as significantly more important than did the younger age cohort. However, over time, the younger age cohort came to rate salvation as increasingly important, until 2002, when the measured percentage starts to be higher in the younger age cohort than in the older. The individuals in the older age cohort start to rate salvation as less important in 2004. In six of the eight years in 1986–1995 (the exceptions are 1991 and 1994), the importance of salvation is significantly higher (*p* < 0.05) among the older age cohort than among the younger. From 1996, the difference between the age cohorts is not statistically significant. Underlying these variations are different valuations of the importance of salvation in the four generations. Merging the data from all investigated years, the Pre-War Generation (22%, n = 9827) and Generation Y (23%, n = 2201) tend to perceive salvation as more important than the Baby Boomers (14%, n = 8843) and Generation X (15%, n = 5016). Over time, as the Pre-War Generation has been replaced by the Baby Boomers, salvation is rated as less important among individuals above 40 years of age. Since Generation X also rates salvation less important than does the Pre-War Generation, the trend toward decreased rated importance of salvation continues when this generation enters the older age cohort. As Hypothesis 3 implies, when the Baby Boomers are replaced with Generation X, and even more so when Generation Y enters the samples, salvation tends to be rated as more important among individuals 40 years of age and younger. In the extremely secular Sweden, Generations X and Y seem to value religious concepts such as salvation more than did the Baby Boomers at a similar age. I claim that this is due to the increasingly deregulated and competitive religious market in Sweden.

Still, lifecycle and period effects, immigration from less secular countries than Sweden, and variations in existential security may explain the generational differences in religious interest. In Table 1, data on the importance of salvation, the years the surveys were conducted, individual age, generation, foreign background, and perception of existential security are compiled in multivariate OLS regressions. The first column presents bivariate OLS estimates. Model 1 includes the Baby Boomers, Generation X and Generation Y (with the Pre-War Generation as the reference category). Model 2 controls for period effects by including the same variables as in Model 1, as well as dummy variables for each survey year (using 2011 as the reference category). Model 3 controls for lifecycle effects and includes the same variables as in Model 1, as well as the age of each individual. Model 4 includes a dummy variable for foreign background together with the same variables as in Model 3. Model 5 includes the existential security index and the same variables as in Model 3. Finally, Model 6 includes all variables from Models 3–5. Since some variables are not included in all survey waves, the number of respondents is lower in Models 4–6.

**Table 1:** Effects of generation, age, period, foreign background, and existential security on the rated importance of salvation; surveys conducted 1986–2011; unstandardized OLS estimates. *Source* SOM Institute, University of Gothenburg

	Bivariate	Model 1^b^	Model 2^b^	Model 3^b^	Model 4^c^	Model 5^d^	Model 6^e^
Constant	–	+2.63***	+2.63***	+1.85***	+1.95***	+2.31***	2.30***
Pre-War Generation	+.41***	–^a^	–^a^	–^a^	–^a^	–^a^	–^a^
Baby Boomers	–.30***	–.42***	–.43***	–.17***	–.21***	–.17***	–.20***
Generation X	–.19***	–.38***	–.38***	+.07*	–.06	–.01	–.07
Generation Y	+.18***	–.06*	–.07**	+.47***	+.35***	+.44***	+.38***
Age	+.01***	–	–	+.01***	+.01***	+.01***	+.01***
Period							
1986	–.14***	–	–.14***	–	–	–	–
1988	+.03	–	+.04	–	–	–	–
1990	–.08**	–	–.08*	–	–	–	–
1991	–.01	–	+.01	–	–	–	–
1992	–.05	–	–.04	–	–	–	–
1993	–.01	–	.00	–	–	–	–
1994	+.16***	–	+.15***	–	–	–	–
1995	–.06**	–	–.05	–	–	–	–
1996	.00	–	+.01	–	–	–	–
1998	+.05	–	+.01	–	–	–	–
2000	+.05*	–	+.05	–	–	–	–
2002	+.15***	–	+.14***	–	–	–	–
2004	.00	–	.00	–	–	–	–
2006	–.08**	–	–.06	–	–	–	–
2008	–.02	–	–.01	–	–	–	–
2011	–.02	–	–^a^	–	–	–	–
Foreign background (foreign = 1, other = 0)	+.39***	–	–	–	+.42***	–	+.35***
Existential security index (insecure = 0, secure = 1)	–.66***	–	–	–	–	–0.65***	–0.59***
Adjusted *R* ^2^	–	.02***	.03***	.03***	.04***	.04***	.04***
*n*	10,821–25,886	25,886	25,886	25,886	17,986	10,708	9257

^a^Reference category

^b^Includes surveys conducted in 1986, 1988, 1990–1996, 1998, 2000, 2002, 2004, 2006, 2008, and 2011

^c^Includes surveys conducted in 1993–1996, 1998, 2000, 2002, 2004, 2006, 2008, and 2011

^d^Includes surveys conducted in 1991, 1993, 1994, 1996, 2006, 2008, and 2011

^e^Includes surveys conducted in 1993, 1994, 1996, 2006, 2008, and 2011

* *p* < 0.10; ** *p* < 0.05; *** *p* < 0.01

The bivariate regression estimates presented in Table 1 support the central findings presented in Fig. 1, since they indicate that the Pre-War Generation attributes salvation as most important while the Baby Boomers attribute it as least important. Of the two younger generations, Generation Y finds salvation more important than does Generation X. In Model 1, the OLS generation estimates indicate that the Baby Boomers and Generation X tend to value salvation significantly less than the Pre-War Generation, while Generation Y values salvation almost as much as the Pre-War Generation. When the possible period effects are controlled for in Model 2, the measured effects of generation hardly change. Contrary to Hypothesis 4, period effects do not change the measured effects of generation on the rated importance of salvation. As Model 3 (as well as the bivariate correlation between age and importance of salvation) shows, people tend to value salvation more with increasing age (Burkimsher 2014; Voas and David 2010). However, Model 3 clearly shows that the estimated generational differences are adjusted for when controlling for age. When age is taken into account, Baby Boomers tend to value salvation less than the Pre-War Generation, Generation X tends to value salvation at the same level as the Pre-War Generation, and Generation Y values salvation more than the Pre-War Generation. This result supports Hypothesis 5—religious interest tends to increase with age, which modifies generational effects on religious interest—and strengthens the empirical support for generational effects on religious interest due to supply changes in the religious market. As Models 4–6 show, even though people of a foreign background perceive salvation as more important than do other people, and even though perceived existential insecurity tends to increase the rated importance of salvation, the OLS generation estimates remain largely intact. However, when controlling for foreign background in Model 4, the values of the OLS estimate related to Generation Y are lower than in Model 3, indicating that, in this generation, interest in salvation is relatively low among Swedes not of a foreign background. Even now, however, the differences between generations are similar to those in Model 3. Since Hypotheses 6 and 7 suggest that foreign background and existential security explain the intergenerational differences in religious interest, they are not supported. This means that the support for Hypothesis 3—when comparing Baby Boomers with Generations X and Y, the younger generations, especially Generation Y, have a greater interested in religion—remains intact.

## Conclusion

The main argument made here—changes in religious supply affect how different generations express their religious demand—is supported. Younger generations express greater religious interest than the older generation of Baby Boomers, contrary to traditional ideas of an ongoing secularization (Bruce 2000). This result supports the proposed theory of religious change in extremely secular societies such as Sweden. Firstly, a free and competitive religious market stimulates interest in religion. Secondly, due to the importance of value formation at a young age and generational replacement, there is a time lag in demand-side responses to changes in religious supply.

The religious deregulation of government policies and informal norms coincided with increased religious pluralism and higher competition in the Swedish religious market. As this study demonstrates, the Swedish religious market changed dramatically around 1970. Compared to older generations, the generations whose formative years were after 1970 experienced an extremely secular society, but at the same time a much freer and more competitive religious market than those of older generations. Since the generations who grew up after the Second World War tend to be more individualized than the Pre-War Generation, during their formative years, the regulated religious market was less suited for the religious demand of Baby Boomers than individuals in the Pre-War Generation. Of the generations in this study, Baby Boomers have the least interest in religion. During the period of religious deregulation and increased competition, Generations X and Y formed their basic interest in religion. As hypothesized, when period and lifecycle effects are controlled for, Generations X and Y, compared with the Baby Boomers, tend to display greater interest in religion, and Generation Y tends to be more interested in religion than Generation X.

When the increased share of individuals of foreign and, in many cases, more religious backgrounds in the Swedish population is accounted for, the intergenerational explanation of religious interest is still valid. In this study, as stated by the external security thesis, people who express existential insecurity tend to value salvation more than do more existentially secure individuals. Even when including existential insecurity, there is no change in how different generations value the importance of salvation. In short, this study supports central assumptions made by the religious market theory. It also underlines the importance to account for a time lag in demand responses to changes of religious supply.

## Footnotes

1 In this study, *religion* refers to belief systems, values and norms, rites, behaviors, and communities related to some transcendent reality that goes beyond natural, physical reality. *Religiosity* is the individual’s subjective perception of a transcendent reality beyond the natural reality (Hagevi 2005).

2
*Individualization* means that individual autonomy in social relations has tended to become more important at the cost of collective identity (Giddens 1991). *Secularization* refers to the declining importance of religion across more sectors of society and to an increasing number of people (Hagevi 2005).

3 Cronbach’s alpha = 0.57. To correct for bias, responses indicating high existential insecurity are pooled so that the maximum value of the index is comprised by at least 1 percent of the responses.
